# Axillary Ectopic Breast Tissue Presenting With Cyclical Swelling: A Case Study

**DOI:** 10.7759/cureus.83618

**Published:** 2025-05-06

**Authors:** Michail Angelos Papaoikonomou, Europi Michailidou, Aggeliki Chlorou

**Affiliations:** 1 School of Medicine, Aristotle University of Thessaloniki, Thessaloniki, GRC; 2 Department of General Surgery, Agios Pavlos General Hospital, Thessaloniki, GRC

**Keywords:** axillary breast tissue, breast development anomalies, breast surgery, ectopic breast tissue (ebt), hormonal changes and ebt

## Abstract

Ectopic breast tissue (EBT) is a rare condition characterized by the presence of abnormal additional breast tissue outside the typical pectoral region, typically along the mammary/milk lines. Although often asymptomatic, EBT can present as a palpable mass in areas such as the axilla, abdomen, or groin and may exhibit cyclical swelling in response to hormonal fluctuations. We report a case of a female patient, age 19, who complained of a palpable tumor in her right axilla. The tumor was linked to sporadic edema that coincided with her menstrual cycle. She had no prior surgical history and denied experiencing any other symptoms, such as discomfort, fever, weight loss, and systemic issues. Ultrasound imaging revealed glandular tissue consistent with EBT. The patient underwent surgical excision of the mass without complication. This case emphasizes the need to include EBT in the differential diagnosis of axillary tumors, especially those with cyclical alterations. Furthermore, it underscores the importance of good imaging in ensuring correct diagnosis and guiding effective treatment, which in this case involved surgical excision of the mass.

## Introduction

Ectopic breast tissue (EBT) is a rare but clinically significant condition that occurs when breast tissue forms or persists outside the normal breast region along the milk line, which typically extends from the axilla to the thigh. During embryogenesis, breast tissue develops along the mammary crest, and in the case of EBT, this tissue may remain or develop abnormally in locations beyond the typical breast area [1]. The axilla is the most common site of ectopic tissue, although it can also be found in the groin, vulva, perineum, and thigh [2]. However, some rare cases of EBT outside the milk line have been reported, such as face, neck, ear, scapula, lateral thigh, knee, foot, vulva, perineum, and buttock [3]. EBT, like normal breast tissue, is influenced by hormonal fluctuations and may develop both benign and malignant lesions, including fibroadenomas, and rarely, breast cancer [4]. The incidence of EBT in women ranges from 2 to 6%, while it occurs in approximately 1-3% of men, often bilaterally, particularly in the axillary region, with multiple sites of supernumerary tissue formation present in about one-third of cases [5]. Kajava's classification system offers a framework for categorizing EBT based on the presence and morphology of glandular tissue, areola, and nipple [5,6]. In this report, the case of a 19-year-old female patient with EBT in the right axilla is classified as Class IV, characterized by the presence of glandular tissue but lacking an areola and nipple. The diagnosis of EBT can be particularly challenging, as its clinical presentation may resemble more common conditions such as sebaceous cysts, hidradenitis, and lymphadenitis, leading to potential misdiagnosis [7]. Although rare, carcinomas in EBT, particularly invasive ductal carcinoma (IDC), can occur, with the axillary region being the most common site of malignancy [4]. This case highlights the importance of considering EBT in the differential diagnosis of axillary masses, as well as the potential for both benign and malignant transformations. Given the possible complications, including infection, ulceration, and malignancy, surgical excision is often recommended to alleviate symptoms and mitigate risks. This report discusses the presentation, diagnostic approach, and management of EBT in a young female patient.

## Case presentation

**Figure 1:**
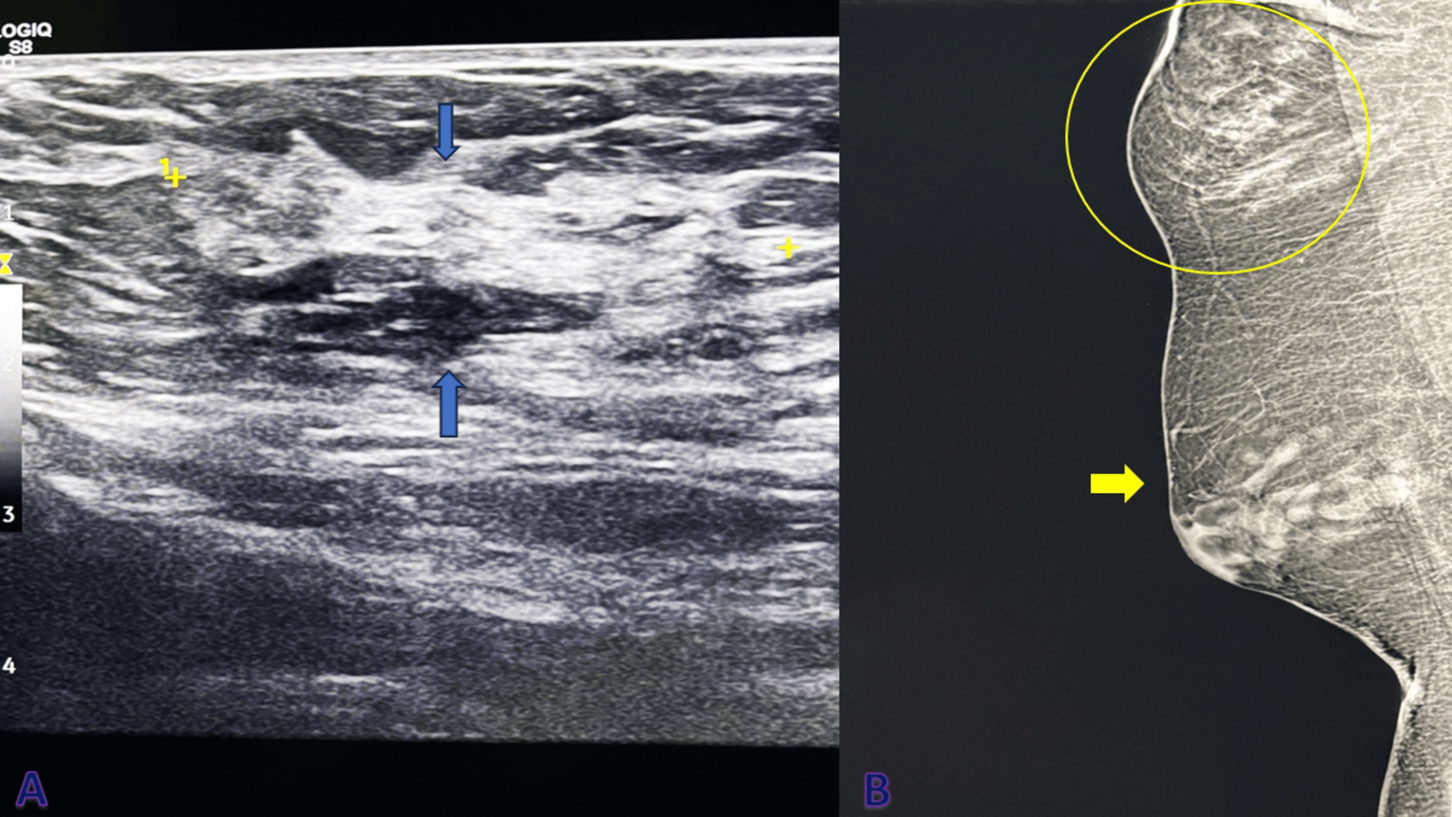
Α. Axillary ultrasound showing a small hypoechoic superficially located mass with irregular margins measuring 3.41 cm (in length which is indicated between the yellow crosses) in the right axilla resembling the features of fibrograndular breast tissue (blue arrows). Β. Appearance of accessory breast tissue on the mammography posterior plane on a mediolateral oblique view of a 19-year-old woman. Asymmetry (circle) is noted on the right, upper hemisphere due to the presence of a lipomatous breast-resembling mass outside the normal breast tissue (yellow arrow).

A 19-year-old woman presented with a complaint of a firm, well-defined, mobile, non-tender mass located in the right axilla. The mass, approximately 3.4 × 4 cm in size, was palpated superficially within the subcutaneous tissue, separated from the parenchyma of the right breast. Notably, no significant lymphadenopathy was observed, and both breasts appeared clinically normal. The patient denied any constitutional symptoms, such as cough, fever, weight loss, and night sweats, although she did report mild cyclic pain, swelling, and discomfort corresponding with her menstrual cycle. The patient is a single individual who does not have any children, and her medical history was unremarkable, with no family history of diabetes, hypertension, cancer, or other significant non-communicable diseases. Apart from occasional dysmenorrhea, her obstetric and gynecologic history was also non-contributory. The patient reported normal monthly menstrual cycles and had no history of using intrauterine contraceptive devices or oral contraceptive pills. Given the clinical presentation and physical findings, an ultrasound examination was performed to further assess the nature of the mass (Figure 1A). The imaging revealed a well-defined, uniformly hypoechoic layer of subcutaneous fat with multiple thin, linear echogenic streaks and small hypoechoic areas, which were consistent with fibroglandular tissue. These findings were suggestive of accessory breast tissue. A mammogram was also performed for educational purposes (Figure 1B). 

**Figure 2:**
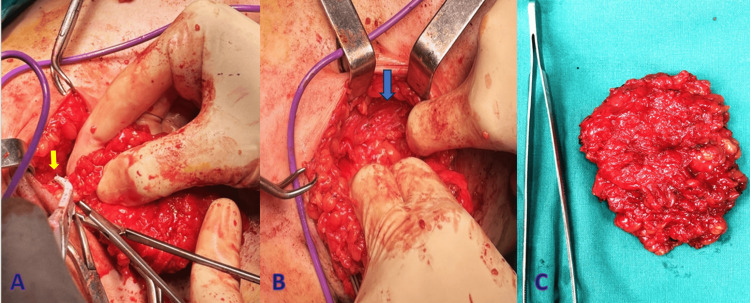
A. Blunt dissection of the axillary accessory breast tissue from the overlying skin with the use of a bipolar electrosurgical instrument (yellow arrow). B. Complete dissection of the mass from the underlying pectoralis major muscle (blue arrow). C. Surgical specimen that was sent for histopathology.

After thorough consultation with the patient, we decided to proceed with surgical excision due to concerns regarding discomfort, aesthetic preferences, and the patient's request for removal. The excision was carried out under general anesthesia. During the procedure, a blunt dissection was performed, and the mass was successfully excised (Figure 2). 

**Figure 3:**
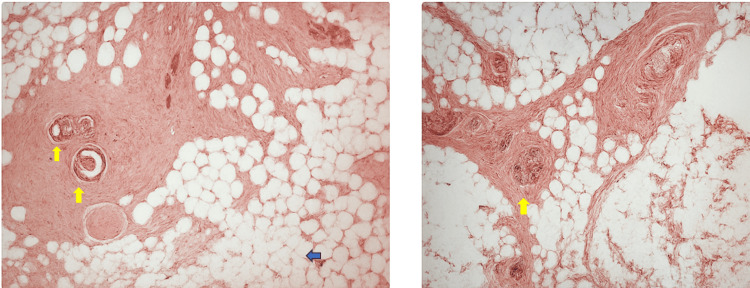
Histological appearance of ectopic axillary breast tissue stained with hematoxylin and eosin (H & E stain) This image demonstrates two sections of ectopic axillary breast tissue. Adipocytes (blue arrow) typical of subcutaneous fat in the axillary region and well-formed ductal structures (yellow arrows), consistent with mammary ducts, encased in fibrous stroma. The ducts exhibit bilayered epithelium, with no atypia or signs of malignancy. These features are characteristic of benign ectopic breast tissue, confirming the diagnosis histologically.

Gross examination of the specimen revealed a soft, lipomatous mass measuring approximately 4 cm in size. The specimen was sent for histological examination. A Penrose drain tube was placed and the wound was closed. Histopathological analysis confirmed the presence of accessory breast tissue, consistent with benign EBT (Figure 3). 

**Figure 4:**
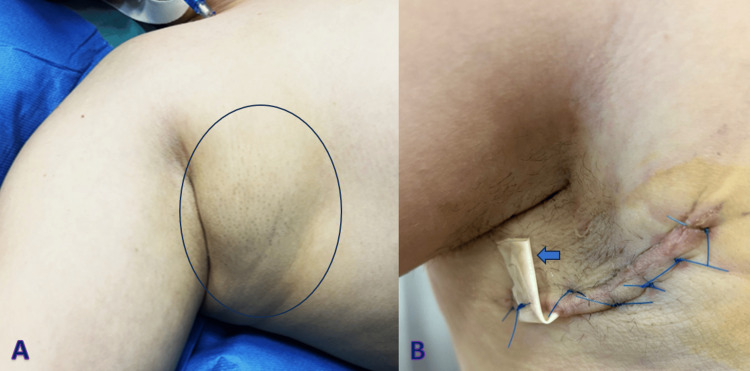
A. Preoperative appearance of the supernumerary axillary breast (blue circle). B. Postoperative appearance of the wound on the 14th postoperative day when the Penrose drain (blue arrow) was removed due to the subsidence of fluid accumulation after the excision showing a smooth aesthetic contour of the patient’s right axilla.

The patient was discharged on the second postoperative day. During the follow-up period, no recurrence or postoperative complications were observed, apart from the anticipated formation of a seroma in the axillary region. This was attributed to the dead space created following the resection, given the substantial size of the EBT. To manage this expected complication, a Penrose drain was placed intraoperatively and was removed on the 14th postoperative day (Figure 4).

## Discussion

This case highlights a rare presentation of EBT in the axilla of a 19-year-old woman, characterized by cyclical swelling and pain correlated with menstruation, and confirmed histologically. While the axilla is the most common site for EBT, the clinical presentation with hormonally responsive symptoms underscores the importance of considering EBT in the differential diagnosis of axillary masses in reproductive-age women. Unlike previously reported cases that often present as incidental or asymptomatic findings, our case emphasizes the functional behavior of EBT mimicking normal breast tissue responsiveness.

EBT, a condition in which breast tissue develops outside the conventional pectoral area, is a rare but important phenomenon in both sexes, more commonly observed in Asian populations [5]. This condition is linked to the mammary ridge, or milk line, which extends from the axilla to the groin during early embryonic development [8]. During the fifth and sixth gestation weeks, the mammary ridge thickens and initiates the development of breast tissue [8,9]. Although this structure typically regresses, incomplete regression may lead to the formation of supernumerary or ectopic breasts along this line [8]. As such, EBT can present with varying degrees of glandular tissue, areolar tissue, and nipple formation, often resulting in cosmetic concerns and potential medical issues [9]. 

Kajava's classification system for EBT categorizes this anomaly into several types based on their constituent tissues, ranging from fully developed breasts (Class I) to abnormal tissue formations like patches of hair (Class VIII) (Table 1) [5,6]. 

**Table 1:** Kajava's classification system for accessory breast tissue. Adapted from references [5,6] under Creative Commons Attribution License and Creative Commons Attribution 4.0 International License

Class	Description
I	Complete breast; includes glandular tissue, nipple, and areola
II	Glandular tissue with nipple; areola absent
III	Glandular tissue with areola; nipple absent
IV	Glandular tissue only; both nipple and areola absent
V	Nipple and areola present; no glandular tissue (pseudomamma)
VI	Nipple only present (polythelia)
VII	Areola only present
VIII	Patch of hair, fat, or skin resembling breast tissue; no true breast elements (pseudomamma)

Τhis classification system for EBT comprises the following elements: Class I is a completely formed breast that includes glandular tissue, the areola, and the nipple; Class II has glandular and nipple tissue but no areola; Class III, which is nipple absent, but areola and glandular tissue present; class IV, which only contains glandular tissue; Class V, which is known as pseudomamma, has the nipple and areola but no glandular tissue; Class VI has extra nipples (polythelia); Class VII only has the areola (polythelia areolaris); and Class VIII only has a patch of hair (polythelia pilosa) [5,6]. EBT typically appears at birth and remains silent until adolescence. The most significant clinical manifestations are those that involve functional glandular tissue capable of lactation, particularly when located in the axilla [9,10]. EBT can be asymptomatic but may cause pain, discomfort, or cosmetic concerns, especially during puberty, pregnancy, or lactation when the ectopic tissue may enlarge or become tender [9, 10]. The differential diagnosis includes sebaceous cyst, fibroadenoma, fibroadenolipoma, hidradenitis suppurativa, lymphadenopathy, vascular malformations, and malignancies (lymphoma and metastatic disease) [3].

The diagnosis of EBT is often clinical, supported by imaging modalities such as mammography, ultrasound, and breast MRI [1]. Sonographically, EBT typically appears as an echogenic area resembling normal glandular tissue [1]. This resemblance to normal breast tissue can sometimes lead to confusion with other entities such as the tail of the breast, lipomas, fibroadenomas, or chest wall tumors, highlighting the importance of accurate imaging and differential diagnosis. A small to large portion of fibroglandular densities scattered with fat that radiographically resemble normal glandular parenchyma but are situated apart from the main breast parenchyma make up the normal mammographic appearance of accessory breast tissue in the axilla [1]. On MRI, EBT might appear as a subcutaneous ill-defined mass or non-mass-like region that has signal intensity and contrast enhancement similar to the rest of the breast parenchyma but is discontinuous with it. Fine-needle aspiration (FNA) cytology plays a key role in distinguishing malignant lesions from benign ones, particularly when a lump is present [11]. In some cases, biopsy and histological examination may reveal benign lesions like fibroadenomas, granulosa cell tumors, or intraductal papillomas, which are commonly associated with EBT. The most common pathology in EBT is benign tumors mainly fibroadenomas [12].

However, EBT's potential for malignancy must not be overlooked. EBT carcinoma accounts for 0.3-0.6% of all breast carcinoma with the axilla being the most common site (58%) [11,12]. Although rare, cases of ectopic breast carcinoma, most commonly IDC, have been documented [4]. Other types of ectopic breast carcinoma such as lobular, mucinous, papillary, medullary and phyllodes carcinoma have also been reported [11,13]. Malignant transformation in EBT may present with similar symptoms to benign lesions, making early detection and histological evaluation critical [11]. Although it can be difficult to differentiate EBT cancer from lymph node metastases from undetected primary lesions, FNA cytology can aid in the diagnosis of malignancy [9,11]. Histologically, the diagnosis of EBT carcinoma is confirmed and lymph node metastases excluded when there is a lack of lymphoid tissue and the adjacent normal breast tissue is present (ducts and lobules) [11]. As in the case of orthotopic breast cancers, testing for estrogen receptor, progesterone receptor, and HER2/neu should be performed on EBT carcinoma while the positivity of the immunohistochemical markers (ER, PR, and gross cystic fluid protein GCDFP-15) helps to distinguish it from adnexal tumors of the axilla, noting that accessory breast tissue and sweat glands develop from the same stem cells [1]. EBT carcinoma is typically treated following the same protocols as conventional breast cancer, including surgery, radiotherapy, and systemic therapy, guided by tumor biology and staging [1,4,11]. The presence of concurrent ipsilateral breast cancer may complicate the staging process but does not necessarily affect the treatment strategy for EBT carcinoma [11].

A plethora of treatment modalities have been documented in the extant literature. The objective of surgical intervention is to minimize the volume of additional axillary breast tissue and to achieve an aesthetically pleasing contour for the axilla. Conventional surgical techniques frequently entail direct excision, liposuction, or a combination of both. The direct excision is performed through a longer incision in the axilla and allows the removal of both adipose and fibroglandular breast tissue, although it often results in significant dead space and disrupts various lymphatic routes, leading to problems with skin re-attachment, as well as the formation of seromas and hematomas [9,13]. Conversely, liposuction is utilized for the treatment of accessory axillary breasts, enabling the extraction of the vast majority of the adipose tissue contributing to contour distortion, with minimal scarring and patient downtime. The most prevalent method of treating EBT is surgical excision, although larger Asian cohorts have recently reported the use of liposuction or excision plus liposuction and microdebrider [9]. The employment of the microdebrider instrument ensures the precise excision of accessory axillary breast tissue, thereby ensuring a sharp and meticulous outcome [13]. This procedure is performed via a single 5-mm incision, which is discreetly located within the axillary skin folds. It provides the operator with the requisite level of control to ensure the precise removal of fibro-glandular breast tissue, thereby restoring an aesthetically pleasing contour to the axilla. At present, no single method has gained widespread acceptance as the standard of care. A Danish study revealed that over 57% of patients treated with surgical excision experienced complications, with seroma (24%) and paraesthesia (17%) being the most common [9]. The resection of EBT has been further associated with complications including residual tissue, wound infection, bleeding, pain, decreased limb mobility, axillary web syndrome and poor wound healing [9]. A significant number of cases of liposuction can frequently result in an incomplete excision, consequently leaving behind a core of breast tissue [14]. In light of these findings, some surgeons have adopted a combination of direct excision and liposuction as a management strategy for cases involving accessory axillary breast tissue [13].

Given the rarity of EBT, particularly in its malignant form, the precise incidence of primary EBT carcinoma and its progression to malignancy remain poorly defined. However, it is essential to maintain a high index of suspicion for EBT carcinoma, particularly in cases where there is a history of concurrent breast cancer, as accurate staging can significantly impact treatment decisions and patient outcomes [8]. Moreover, EBT's low prevalence and the reluctance of patients to seek regular screening for this condition complicate efforts to gather more comprehensive data on its natural history and association with other breast diseases [15].

This study has certain drawbacks. This is an empirical narrative of an ectopic breast occurrence that cannot be applied to all cases. There is a pressing need for further investigation into the clinical course, imaging characteristics, and histopathology of pathologies found in EBT. Such research will aid in refining diagnostic criteria, improving early detection, and enhancing the management of this unique and potentially problematic condition.

## Conclusions

In conclusion, while EBT is typically benign, it remains a clinically relevant entity due to its potential for malignancy and its ability to cause physical, cosmetic, and emotional distress. A multidisciplinary approach to diagnosis and treatment, including careful imaging, clinical evaluation, and histological confirmation, is essential to ensuring optimal patient outcomes. This case report raises clinical awareness by illustrating the clinical importance of axillary swelling, including EBT, which experiences physiological alterations analogous to those of orthotopic breast tissue. Due to the rarity of the condition, more documented cases are needed to understand potential risks (e.g., malignant transformation), optimal management, and long-term follow-up strategies. This report contributes valuable data toward that goal.
